# Supplementary material for: A neural mechanism for contextualizing fragmented inputs during naturalistic vision
Source: eLife. 2019 Oct 9;8:e48182. doi: 10.7554/eLife.48182 (PMC6802952; doi:10.7554/eLife.48182)
Supplement: Supplementary file 1. — The table shows test statistics and p-values for all tests performed in the fMRI experiment (Figures 2 and 3). Values reflect one-sided t-tests against zero. All p-values are uncorrected; in the main manuscript, only tests surviving Bonferroni-correction across the three ROIs (marked in color) are considered significant. [file elife-48182-supp1.docx]

| V1 | vertical location | horizontal location | category |
| --- | --- | --- | --- |
| original analysis  (Fig. 2d) | t[29]=1.63  p=0.057 | t[29]=-0.27  p=0.61 | t[29]=1.18  p=0.12 |
| DNN removed  (Fig. 3b) | t[29]=-0.21  p=0.58 | t[29]=-0.55  p=0.71 | t[29]=1.02  p=0.16 |
| within-type removed  (Fig. 3e) | t[29]=1.49  p=0.074 | t[29]=-0.49  p=0.69 | N/A |
| both removed  (Fig. 3h) | t[29]=-0.08  p=0.53 | t[29]=-0.79  p=0.78 | N/A |
| OPA | **vertical location** | **horizontal location** | **category** |
| original analysis  (Fig. 2d) | t[29]=4.12  p<0.001 | t[29]=1.31  p=0.10 | t[29]=3.12  p=0.002 |
| DNN removed  (Fig. 3b) | t[29]=2.37  p=0.012 | t[29]=1.08  p=0.14 | t[29]=1.29  p=0.10 |
| within-type removed  (Fig. 3e) | t[29]=3.05  p=0.002 | t[29]=1.27  p=0.11 | N/A |
| both removed  (Fig. 3h) | t[29]=2.38  p=0.012 | t[29]=0.93  p=0.18 | N/A |
| PPA | **vertical location** | **horizontal location** | **category** |
| original analysis  (Fig. 2d) | t[29]=2.03  p=0.026 | t[29]=0.34  p=0.37 | t[29]=4.26  p<0.001 |
| DNN removed  (Fig. 3b) | t[29]=0.002  p=0.50 | t[29]=0.11  p=0.46 | t[29]=1.34  p=0.10 |
| within-type removed  (Fig. 3e) | t[29]=1.61  p=0.059 | t[29]=0.19  p=0.43 | N/A |
| both removed  (Fig. 3h) | t[29]=0.10  p=0.46 | t[29]=0.003  p=0.50 | N/A |
