# Supplementary material for: A neural mechanism for contextualizing fragmented inputs during naturalistic vision
Source: eLife. 2019 Oct 9;8:e48182. doi: 10.7554/eLife.48182 (PMC6802952; doi:10.7554/eLife.48182)
Supplement: Supplementary file 2. — The table shows means and standard deviations (in brackets) of peak latencies in ms for vertical location and category information in the main analyses (Figures 2 and 3). To estimate the reliability of peaks and onsets (Supplementary file 3) of location and category information in the key analyses, we conducted a bootstrapping analysis. For this analysis, we choose 100 samples of 20 randomly chosen datasets (with possible repetitions). For each random sample, we computed peak and onset latencies; we then averaged the peak and onset latencies across the 100 samples. Peak latencies were defined as the highest beta estimate in the time course. Notably, the peak latency of vertical location information remained highly stable across analyses. [file elife-48182-supp2.docx]

|  | vertical location | vertical location  (across indoor/outdoor) | category |
| --- | --- | --- | --- |
| original analysis | 215 (15) | 214 (25) | 348 (165) |
| DNN removed | 219 (20) | 219 (47) | *n.s.* |
