# Supplementary material for: A neural mechanism for contextualizing fragmented inputs during naturalistic vision
Source: eLife. 2019 Oct 9;8:e48182. doi: 10.7554/eLife.48182 (PMC6802952; doi:10.7554/eLife.48182)
Supplement: Supplementary file 3. — The table shows means and standard deviations (in brackets) of onset latencies in ms for vertical location and category information in the main analyses (Figures 2 and 3)). Onset latencies were quantified using the bootstrapping logic explained above (Supplementary file 2). Onsets were defined by first computing TFCE statistics for each random sample, with multiple-comparison correction based on 1000 null distributions. The onset latency for each sample was then defined as the first occurrence of three consecutive time points reaching significance (p<0.05, corrected for multiple comparisons). [file elife-48182-supp3.docx]

|  | vertical location | vertical location  (across indoor/outdoor) | category |
| --- | --- | --- | --- |
| original analysis | 62 (8) | 84 (16) | 62 (4) |
| DNN removed | 98 (36) | 170 (46) | *n.s.* |
